# The Methods of Lymph Node Examination Make a Difference to Node Staging and Detection of N3b Node Status for Gastric Cancer

**DOI:** 10.3389/fonc.2020.00123

**Published:** 2020-02-12

**Authors:** Xinhua Chen, Yuehong Chen, Yanfeng Hu, Tian Lin, Jun Luo, Tuanjie Li, Tao Li, HuiLin Huang, Yu Zhu, Tingting Li, Hao Chen, Hao Liu, Guoxin Li, Jiang Yu

**Affiliations:** Department of General Surgery, Nanfang Hospital, Southern Medical University, Guangzhou, China

**Keywords:** gastric cancer, lymph node, examination, node staging, N3b

## Abstract

**Background:** The number of retrieved lymph nodes (RLNs) affects the likelihood of detecting metastatic lymph nodes (MLNs) for gastric cancer (GC), but the retrieval of LNs is not satisfactory worldwide. There is no standard for LN examination.

**Methods:** We retrospectively analyzed 2,163 patients diagnosed with GC who underwent surgery at Nanfang Hospital between October 2004 and September 2016. According to the methods of LN examination, patients were classified into two groups: LN detection by pathologists (pathologist group) and LN examination by surgicopathologic team (surgicopathologist group). The relationship between RLNs and LN staging accuracy as well as the factors influencing the detection of MLNs were evaluated.

**Results:** There were 472 males in pathologist group and 467 males in surgicopathologist group. The number of RLNs and MLNs in surgicopathologist group was significantly higher than that in pathologist group (RLNs: 53.8 ± 20.9 vs. 18.8 ± 11.5, *p* < 0.001; MLNs: 5.6 ± 9.8 vs. 3.9 ± 5.7, *p* < 0.001). Notably, the detection of N3b node status was significantly improved in surgicopathologist group [83 (11.9%) vs. 34 (4.8%), *p* < 0.001]. Additionally, the detection rate of N3b status gradually increased from 0 in patients with 1-16 RLNs to 16.6% in patients with more than 49 RLNs. The MLNs detected increased gradually from 2.3 ± 3.0 in patients with 1-16 RLNs to 7.3 ± 11.7 in patients with more than 49 RLNs. Univariate and multivariate analyses indicated that LN examination by surgicopathologic team, more advanced pT, tumor size ≥5 cm and combined organ(s) resection were related to detecting more MLNs.

**Conclusions:** The retrieval of nodes immediately postoperatively by the surgicopathologic team could significantly improve the number of RLNs, detect more MLNs, and screen more patients with N3b node status.

## Introduction

Many studies have suggested that overall survival (OS) is associated with the number of retrieved lymph nodes (RLNs) (1–3). The results based on data from the Surveillance, Epidemiology, and End Results (SEER) database showed that OS was dependent on the number of RLNs (1). For every 10 additional LNs examined, all four stage subgroups could yield superior survival, and this tendency could continue to be detected for cutoff points of up to 40 LNs. However, the number of RLNs cannot separate the impact of stage migration versus improved regional disease control to favor survival. Recently, Hayashi et al. showed that the number of RLNs < 40 could be attributed to an inferior survival for stage III gastric cancer (GC) patients who underwent total gastrectomy (2). Consistently, another large international dataset analysis, including the SEER database (*n* = 13,932) and the Yonsei University Gastric Cancer database (*n* = 11,358), also proposed that a greater number of RLNs (a minimum of 29) improves staging and OS in GC patients undergoing radical resection (3). All of these quality studies proposed a higher number of RLNs than that recommended by the 8th edition of the American Joint Committee on Cancer (AJCC) TNM staging system for GC (at least 16).

To determine why OS following operations for GC in Japan are far superior to the results obtained in Western countries, Noguchi et al. reviewed the Japanese literature and found that the meticulous histopathological evaluation of surgical specimens in Japan resulted in more accurate pathologic staging, which was one of the main reasons for the improved OS (4). Consistent with this finding, many subsequent studies also demonstrated that the number of RLNs could affect the likelihood of detecting metastatic LNs (MLNs) (5) and stage migration (6–8).

For standard D2 lymphadenectomy, which can guarantee the efficiency of locoregional disease control for resectable GC, the number of RLNs is mainly dependent on the approach of LN examination. Furthermore, the results of the retrieval of LNs in the Dutch Gastric Cancer Trial suggest that LN retrieval rather than the extent of surgical LN dissection was mainly responsible for the number of RLNs (9).

Taken together, these findings suggest that the improper approach of LN examination could result in the insufficiency of RLNs and the underestimation of LN metastasis status, which could have an undesirable impact on prognostic evaluation and the strategy formulation of adjuvant therapy. However, Sano et al. collected analytic data on 25,411 patients from 59 institutions in 15 countries, showing that the mean/median (range) number of LNs examined in Japan, Korea, selected other Asian centers and selected Western centers was 39.4/36 (1–171), 33/31 (1–129), 24.8/22 (1–103), and 29.5/27 (1–123), respectively (10).

Therefore, more standard and normative LN examination techniques are urgently needed, as well as the identification of confounding factors in nodal status assessment, to further improve the accuracy of node assessment and therefore improve survival (7, 8, 11, 12). Hence, we summarize the methods and experiences of LN examination for GC specimens at the Department of General Surgery of Nanfang Hospital by comparing the nodal yields obtained by conventional sampling by pathologists vs. immediate postoperative retrieval by the surgicopathologic team.

## Methods

### Patients

**Figure 1:**
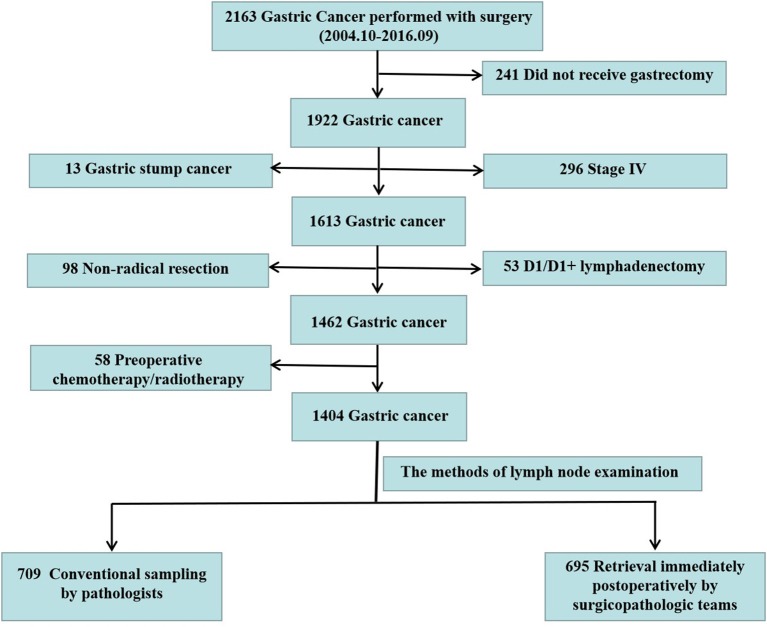
Flow chart of the study cohort.

In the period between October 2004 and September 2016, 2,163 consecutive patients were diagnosed with GC and underwent surgery at Nanfang Hospital, Southern Medical University. The analyses were based on the prospective GC database, which includes information on GC derived from electronic medical records that have been maintained in the Nanfang Hospital since 2004 (13). Data monitoring was performed by a specific medical recorder with ~10 years of relevant work experience. The recorded variables included demographic, clinical, pathological, and surgical characteristics. After two independent surgical oncologists reviewed the pathological reports and medical records of the patients retrospectively, patients who did not receive gastrectomy, underwent non-radical resection, had stage IV GC, underwent only D1/D1+ lymphadenectomy rather than D2/D2+ lymphadenectomy, had gastric stump cancer or had neoadjuvant chemotherapy before gastrectomy were excluded. After the above exclusion criteria were evaluated, 1,404 patients were enrolled. According to the methods of LN examination, patients were classified into two groups: conventional method for retrieving LNs by pathologists (pathologist group) and standard operating procedure (SOP) of LN examination by a specialized surgicopathologic team (surgicopathologist group) (Figure 1). For patients in the pathologist group, the pathologists retrieved LNs after the specimens were fixed in 10% neutral buffered formalin. For patients in surgicopathologist group, a member of the surgicopathologic team sequentially retrieved LNs postoperatively within 5 min according to the LN station and then submitted the LN specimens to the Pathology Department for further examination.

The cancer stage was determined or recoded according to the 7th edition of the AJCC TNM staging system (13). Tumor location was categorized as the upper, middle, or lower third of the stomach. The resection approach [laparoscopic gastrectomy (LG) or open gastrectomy (OG)] and reconstruction methods followed standard guidelines (14–17). The study complied with the principles set forth in the Declaration of Helsinki. The data collection protocol was approved by the Ethics Committee of Nanfang Hospital, Southern Medical University. Written informed consent was obtained from all the patients in the study.

### LN Examination Approaches

#### Pathologist Group

LN retrieval was conventionally performed by pathologists via inspection, palpation, and/or serial sectioning of the formalin-fixed specimens.

#### Surgicopathologist Group

The practice of LN examination by the specialized surgicopathologic team included three parts (18): the establishment of a special team (the surgicopathologic team) to examine LNs, the development of an effective SOP for LN examination, and long-term and sustained quality control. The special team was composed of postgraduate students who were not involved in surgery but trained professionally by surgeons. The SOP includes studying the anatomy of the perigastric region, learning surgical procedures to identify LN stations and specifying procedures for LN examination. The specification procedures in more detail are showed in Appendix. Last, quality control consisted of periodic data reporting and continuous feedback to ensure the high quality of the LN examination since personnel changes occurred often.

### Statistical Analysis

Data are presented as the mean ± standard deviation (SD) for continuous variables (for variables with non-normal distributions, the medians and ranges are shown) and as numbers (%) for categorical variables. Student's *t* test and the Mann-Whitney *U* test were used to compare continuous variables, and the χ^2^ test and Fisher's exact test were used to compare categorical variables as appropriate. Risk factors for the number of MLNs were evaluated by univariate analyses and multivariate analyses using a general linear regression model. *p* < 0.05 (two-tailed) was considered statistically significant. The statistical software SPSS version 17.0 for Windows (SPSS, Inc., Chicago, IL, USA) was used for all statistical analyses.

## Results

### Patient Characteristics

The clinical and pathological characteristics of the patients are shown in Table 1. There were 472 males in the pathologist group and 467 males in the surgicopathologist group (*p* = 0.805). The mean age in the pathologist group and surgicopathologist group was 55.7 and 56.1 years (*p* = 0.485), with a mean body mass index of 21.6 and 22.5 kg/m^2^ (*p* = 0.030), respectively. The clinical depth of tumor invasion (cT) was more advanced in patients in the pathologist group than in those in the surgicopathologist group [cT1/cT2/cT3/cT4: 124(17.5%)/60(8.5%)/110(15.5%)/415(58.5%) vs. 53(7.6%)/74(10.6%)/101(14.5%)/467(67.2%), *p* < 0.001]. Similarly, pathological tumor depth (pT) was also increased in patients in the pathologist group than in those in the surgicopathologist group [pT1a/pT1b/pT2/pT3/cT4a/pT4b: 61(8.6%)/61(8.6%)/80(11.3%)/15(2.1%)/430(60.6%)/62(8.7%)vs. 71(10.2%)/88(12.7%)/69(9.9%)/111(16.0%)/309(44.5%)/47(67.6%), *p* < 0.001]. Nevertheless, although the clinical LN status (cN) was more advanced in the pathologist group [cN0/cTN1/cN2/cN3: 220(31.0%)/119(16.8%)/246(34.7%)/124(17.5%) vs. 330(47.5%)/143(20.6%)/108(15.5%)/114(16.4%), *p* < 0.001], the pathological LN status (pN) was not significantly different between the two groups [pN0/pTN1/pN2/pN3a/pN3b: 263(37.1%)/135(19.0%)/166(23.4%)/111(15.7%)/34(4.8%) vs. 328(47.2%)/92(13.2%)/86(12.4%)/106(15.3%)/83(11.9%), *p* = 0.248]. Consistent with the pT status, the patients in the pathologist group more likely underwent gastrectomy with combined organ(s) resection [60(8.5%) vs. 40(5.8%), *p* = 0.049]. Patients in the pathologist group were more inclined to undergo open surgery [345(48.7%) vs. 83(11.9%), *p* < 0.001] and distal gastrectomy [511(72.1%) vs. 441(63.5%), *p* < 0.001], with less surgery time [surgery time ≥240 min: 122(17.2%) vs. 210(30.2%), *p* < 0.001] but more blood loss [estimated blood ≥400 ml: 99(14.0%) vs. 37(5.3%), *p* < 0.001]. There were no significant differences between the pathologist group and the surgicopathologist group in terms of tumor size, number of primary lesions, or comorbidity of diabetes.

**Table 1:** Clinicopathologic characteristics of the two groups of patients.

**Characteristic**	**Total (*n* = 1,404)**	**Pathologist group (*n* = 709)**	**Surgico-pathologist group (*n* = 695)**	**Statistic**	***p-value***
Gender [*n* (%)]				0.061	0.805
Male	939 (66.9)	472 (66.6)	467 (67.2)		
Female	465 (33.1)	237 (33.4)	228 (32.8)		
Age, y, mean ± SD		55.7 ± 12.0	56.1 ± 11.7	−0.699	0.485
Body mass index, mean ± SD		21.6 ± 3.0	22.5 ± 8.1	−2.178	0.030
Diabetes [*n* (%)]				0.345	0.557
Yes	66 (4.7)	31 (4.4)	35 (5.0)		
No	1,338 (95.3)	678 (95.6)	660 (95.0)		
Tumore size [*n* (%)]					0.793
<5 cm	940 (67.0)	477 (67.3)	463 (66.6)		
≥5 cm	464 (33.0)	232 (32.7)	232 (33.4)		
cT-stage [*n* (%)]				−4.062	<0.001
cT1	177 (12.6)	124 (17.5)	53 (7.6)		
cT2	134 (9.5)	60 (8.5)	74 (10.6)		
cT3	211 (15.0)	110 (15.5)	101 (14.5)		
cT4	882 (62.8)	415 (58.5)	467 (67.2)		
pT-stage [*n* (%)]				−5.195	<0.001
pT1a	132 (9.4)	61 (8.6)	71 (10.2)		
pT1b	149 (10.6)	61 (8.6)	88 (12.7)		
pT2	149(10.6)	80(11.3)	69(9.9)		
pT3	126 (9.0)	15 (2.1)	111 (16.0)		
pT4a	739 (52.6)	430 (60.6)	309 (44.5)		
pT4b	109 (7.8)	62 (8.7)	47 (67.6)		
cN stage [*n* (%)]				−6.403	<0.001
cN0	550 (39.2)	220 (31.0)	330 (47.5)		
cN1	262 (18.7)	119 (16.8)	143 (20.6)		
cN2	354 (25.2)	246 (34.7)	108 (15.5)		
cN3	238 (17.0)	124 (17.5)	114 (16.4)		
pN stage [*n* (%)]				−1.155	0.248
pN0	591 (42.1)	263 (37.1)	328 (47.2)		
pN1	227 (16.2)	135 (19.0)	92 (13.2)		
pN2	252 (17.9)	166 (23.4)	86 (12.4)		
pN3a	217 (15.5)	111 (15.7)	106 (15.3)		
pN3b	117 (8.3)	34 (4.8)	83 (11.9)		
No. lesions [*n* (%)]				0.543	0.461
Single	1,385 (98.6)	701 (98.9)	684 (98.4)		
Multiply	19 (1.4)	8 (1.1)	11 (1.6)		
Approach [*n* (%)]				223.282	<0.001
Open	428 (30.5)	345 (48.7)	83 (11.9)		
Laparoscopy	976 (69.5)	364 (51.3)	612 (88.1)		
Gastrectomy [*n* (%)]				11.947	0.001
Distal	952 (67.8)	511 (72.1)	441 (63.5)		
Total	452 (32.2)	198 (27.9)	254 (36.5)		
Combined organ(s) resection [*n*(%)]				3.888	0.049
Yes	100 (7.1)	60 (8.5)	40 (5.8)		
No	1,304 (92.9)	649 (91.5)	655 (94.2)		
Surgery time [*n* (%)]				32.894	<0.001
<240 min	1,072 (76.4)	587 (82.8)	485 (69.8)		
≥240 min	332 (23.6)	122 (17.2)	210 (30.2)		
Blood loss [*n* (%)]				29.945	<0.001
<400 ml	1,268 (90.3)	610 (86.0)	658 (94.7)		
≥400 min	136 (9.7)	99 (14.0)	37 (5.3)		

### Effect of the LN Examination Approach on the Number of RLNs and MLNs and the Detection of N3b Status

**Figure 2:**
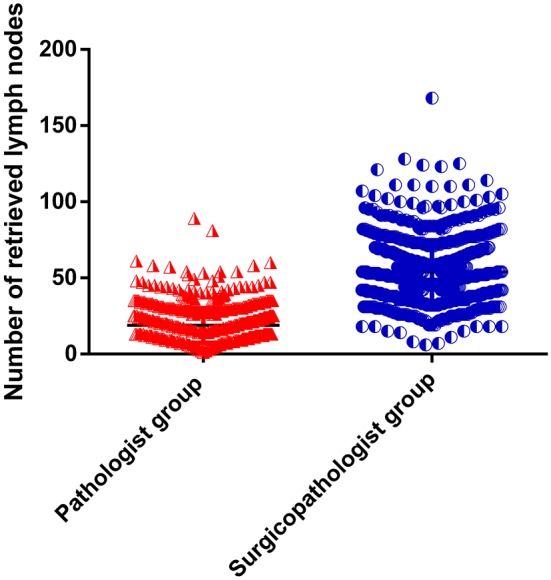
The number of retrieved lymph nodes between the pathologist group and surgicopathologist group.

**Figure 3:**
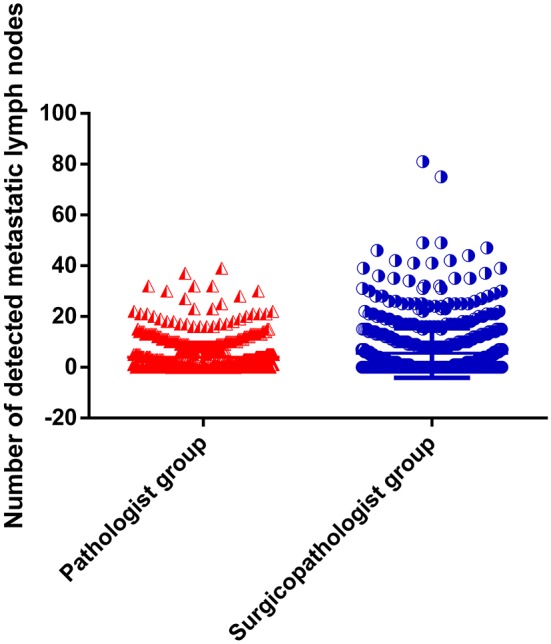
The number of detected metastatic lymph nodes between the pathologist group and surgicopathologist group.

**Figure 4:**
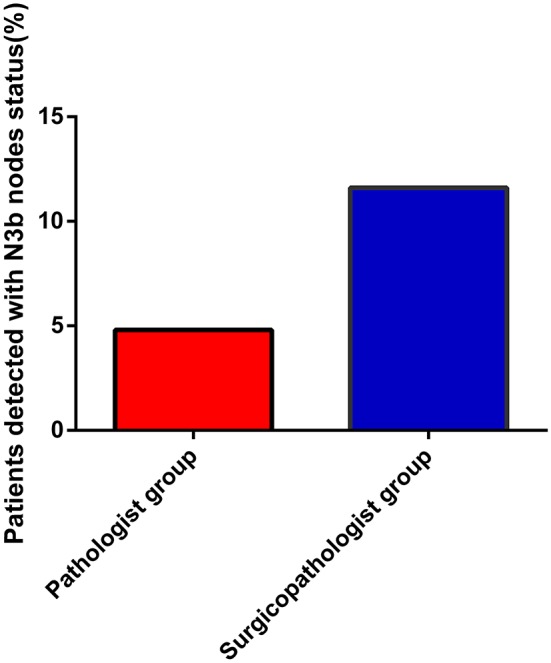
The rate of detecting N3b status between the pathologist group and surgicopathologist group.

As shown in Table 2, the mean number of RLNs in the surgicopathologist group was significantly higher than that in the pathologist group (18.8 ± 11.5 vs. 53.8 ± 20.9, *p* < 0.001) (Figure 2); the same trend was observed in the mean number of MLNs between two groups (3.9 ± 5.7 vs. 5.6 ± 9.8, *p* < 0.001) (Figure 3). More importantly, the detection of N3b node status was significantly improved in the surgicopathologist group [34(4.8%) vs. 83(11.9%), *p* < 0.001] (Figure 4).

**Table 2:** The number of retrieved lymph nodes and metastatic lymph nodes and the dectecting of N3b status in the two groups.

**Variables**	**Total (*n* = 1,404)**	**Pathologist group (*n* = 709)**	**Surgicopathologist group (*n* = 695)**	**Statistic**	***p-value***
RLNs^*^, mean ± SD		18.8 ± 11.5	53.8 ± 20.9	−38.788	<0.001
MLNs^#^, mean ± SD		3.9 ± 5.7	5.6 ± 9.8	−3.917	<0.001
N3b status [*n* (%)]				−4.843	<0.001
N3b	117 (8.3)	34 (4.8)	83 (11.9)		
Non-N3b	1,287 (91.7)	675 (95.2)	612 (88.1)		

* *RLNs, retrieved lymph nodes*.

# *MLNs, metastatic lymph nodes*.

### Effect of the Number of RLNs on MLNs and the Detection of N3b Status

The relationship between the number of MLNs and the number of RLNs and the association between the detection of N3b nodes and the number of RLNs are shown in Table 3. With the increase in RLNs, the number of MLNs also increased [1–16 RLNs vs. 17–32 RLNs vs. 33–48 RLNs vs. >49 RLNs: 2.3 ± 3.0 vs. 4.3 ± 6.1 vs. 4.6 ± 7.0 vs. 7.3 ± 11.7, *p* < 0.001] (Figure 5). In addition, the detection of N3b nodes was also dependent on the number of RLNs [1–16 RLNs vs. 17–32 RLNs vs. 33–48 RLNs vs. >49 RLNs: 0 vs. 25(6.9%) vs. 24(8.7%) vs. 68(13.9%), *p* < 0.001].

**Table 3:** The number of metastatic lymph node and the dectecting of N3b status in the four groups with different retrieved lymph node.

RLNs^*^	**1–16 LNs (*n* = 355)**	**17–32 LNs (*n* = 364)**	**33–48 LNs (*n* = 276)**	**≥49 LNs (*n* = 409)**	**Statistic**	***p-value***
MLNs^#^, mean ± SD	2.3 ± 3.0	4.3 ± 6.1	4.6 ± 7.0	7.3 ± 11.7	26.414	<0.001
N3b stage [*n* (%)]					70.162	<0.001
N3b	0 (0)	25 (6.9)	24 (8.7)	68 (16.6)		
Non-N3b	355 (100)	339 (93.1)	252 (91.3)	341 (69.6)		

* *RLNs, retrieved lymph nodes*.

# *MLNs, metastatic lymph nodes*.

**Figure 5:**
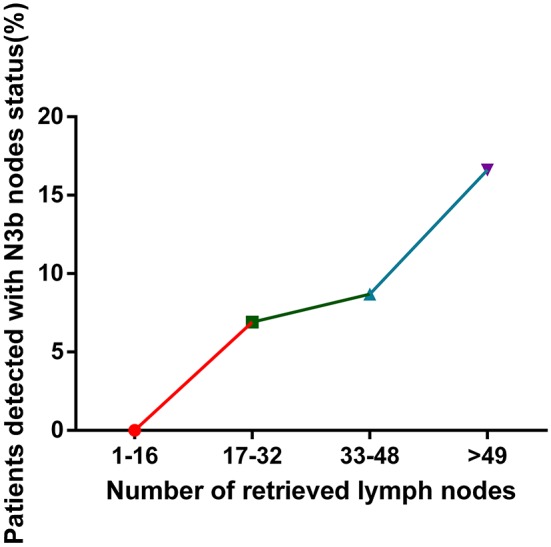
Relationship between the number of retrieved lymph nodes and the rate of the detection of N3b status.

### Factors Influencing the Detection of MLNs

As shown in Table 4, univariate analyses revealed that the method of LN examination, pT, tumor size, and combined organ resection were related to the number of detected MLNs. Furthermore, multivariate analyses indicated that LN examination by the specialized surgicopathologic team, more advanced pT, tumor size ≥5 cm, and combined organ(s) resection were associated with detecting a greater number of MLNs.

**Table 4:** Univariate and multivariate analyses of factors influencing the detecting of metastatic lymph node in this cohort.

**Variables**	**MLNs^#^ (x ± s)**	**Univariate analyses**		**Multivariate analyses**	
		**Mean square**	***p***	**Mean square**	***p***
Approach of LN examination		989.3	<0.001	848.7	<0.001
By pathologists	3.9 ± 5.7				
By surgicopathologists	5.6 ± 9.8				
Gender		90.1	0.237	0.6	0.919
Male	4.9 ± 8.5				
Female	4.4 ± 7.1				
Age		10.2	0.691	28.2	0.479
<65 years	4.7 ± 8.1				
≥65 years	4.9 ± 7.7				
Body mass index		5.0	0.782	7.9	0.707
<28 kg/m^2^	4.7 ± 8.0				
≥28 kg/m^2^	7.7 ± 1.3				
Diabetes		30.2	0.494	48.6	0.352
Yes	5.4 ± 8.7				
No	4.7 ± 8.0				
pT stage		1772.4	<0.001	1191.7	<0.001
pT1a	0.1 ± 0.7				
pT1b	1.2 ± 3.7				
pT2	2.0 ± 3.6				
pT3	5.3 ± 10.6				
pT4a	6.3 ± 8.6				
pT4b	8.2 ± 9.3				
No. of lesion		58.9	0.339	26.4	0.493
Single	4.8 ± 8.0				
Multiply	3.0 ± 9.6				
Tumor size [*n* (%)]		4484.7	<0.001	1472.4	<0.001
<5 cm	3.5 ± 7.1				
≥5 cm	7.3 ± 9.2				
Approach [*n* (%)]		676.7	<0.001	90.5	0.204
Open	3.7 ± 5.6				
Laparoscopy	5.2 ± 8.9				
Gastrectomy [*n* (%)]		2121.3	<0.001	61.8	0.294
Total	6.5 ± 9.1				
Distal	3.9 ± 7.3				
Combined organ(s) resection [*n* (%)]		1.1	0.897	242.3	0.038
No	4.7 ± 8.1				
Yes	4.9 ± 7.0				
Surgery time		983.2	<0.001	141.2	0.113
<240 min	4.3 ± 7.3				
≥240 min	6.3 ± 9.9				
Blood loss		0.212	0.954	3.690	0.798
<400 ml	4.8 ± 8.1				
≥400 ml	4.8 ± 7.1				

# *MLNs, metastatic lymph nodes*.

## Discussion

At most GC centers, LNs are retrieved by pathologists, especially in Western countries and in China. However, pathologists usually describe only the positive and total numbers of greater and lesser curvature LNs. By comparing the significant disparity in nodal yields between surgeons and pathologists, Bunt et al. proposed that the retrieval of LNs should be performed immediately postoperatively by surgeons (9). In their opinion, the following factors may contribute to the essential higher nodal yielding obtained by surgicopathologic teams compared with pathologists: (1) better knowledge of the locations of LNs; (2) more experienced with and dedication to the mission of retrieving more LNs (19, 20); and (3) immediate postoperative processing of the fresh specimen without being fixed by formalin, which facilitates the detection of LNs due to the differences in consistency from fatty tissue. LN retrieval from the operation specimen not by pathologists but by the surgicopathologic team (who have been trained with anatomical and surgical learning programs) can obtain a better three-dimensional view of the anatomical relationships. Furthermore, previous studies have demonstrated that despite some anatomical variability in the distribution of LNs, LN retrieval by the surgicopathologic team, rather than by the pathologists, could lead to more RLNs, which is helpful for standardizing the nodal status assessment (9, 21). Consistently, in our trial, the number of RLNs in the surgicopathologist group was significantly higher than that in the pathologist group (18.8 ± 11.5 vs. 53.8 ± 20.9, *p* < 0.001); the surgicopathologist group also detected a greater number of MLNs (3.9 ± 5.7 vs. 5.6 ± 9.8, *p* < 0.001). More importantly, our trial evaluated the impact of the LN examination approach and the number of RLNs on the N stage assessment, especially the N3b stage, which has not yet been investigated in other similar studies. In our study, patients in the pathologist group had more advanced cT and subsequent pT. Nevertheless, although the cN status was more advanced in the pathologist group, the pN status was not significantly different between the two groups. These results contradict the fact that the more advanced the depth of tumor invasion is, the more advanced the LN status becomes in GC (22–25). Since our trial excluded patients with preoperative chemotherapy or D1/D1+ gastrectomy, we speculated that the inconsistency between cN and pN could be attributed to the methods of LN examination. Additionally, the detection of N3b node status was significantly improved in the surgicopathologist group [34(4.8%) vs. 83(11.9%), *p* < 0.001]. Notably, the N3b status was first put forward by the 7th AJCC TNM staging system in 2014, and the 8th AJCC edition incorporated it into the TNM stage for the first time. The International Gastric Cancer Association (IGCA) Project study, which analyzed the clinical and pathological data of 25,441 patients from 15 countries and 53 institutions who underwent curative gastrectomy, demonstrated that the N3a, and N3b subgroups significantly differed in terms of the 5-year survival rate (10). On the basis of this analysis, the 8th edition AJCC attached great importance to the impact of N3b on the TNM stage. Even in early GC, the N3b node status (T1N3b) could classify patients as stage IIIB, while the T1N0, T1N1, T1N2, and T1N3a were classified as only stage IA, IB, IIA, and IIB, respectively. It showed that N3b node status could have a great impact on disease stage. Thus, the N3b subgroup should be particularly evaluated. A study based on a Chinese cohort also confirmed this phenomenon (26). In this study, N3b patients, regardless of the depth of tumor invasion, exhibited late-stage disease. Sun et al. even classified T4N3b as stage IV (27). Some fundamental research has supported the phenomenon of LN metastasis extension in clinical practice. Recently, a study conducted by Massachusetts General Hospital (MGH) showed that cancer cells from metastatic LNs can escape into the circulation and become the main source of cancer cells for distant metastasis in mouse models (28). The same conclusion was independently obtained at Medical University of Vienna using different methodologies at almost the same time (29). These findings are helpful in providing clues to the clinical significance of N3b and provide implications for facilitating a decision regarding the subsequent use of radiotherapy and chemotherapy treatment and predicting prognosis. Since all N3b patients are classified as stage IIIA or IIIB according to the 8th edition AJCC, the detection of N3b could be used to identify more high-risk stage III GC patients, which is important regarding adjuvant treatment. The positive result of the phase III trial the Adjuvant Chemotherapy Trial of S-1 for Gastric Cancer (ACTS-GC) laid the foundation of ACT for patients with stage II and III GC who had undergone D2 surgery with the regimen of a postoperative S-1 single-agent for 12 months (30). However, subgroup analysis found that the 5-year OS rate of stage IIIB GC patients was 50.2% in the group receiving S-1 after surgery and 44.1% in the group receiving surgery only (HR, 0.791; 95% CI, 0.520–1.205), indicating that there is still some room for improvement. Recently, the Japan Clinical Cancer Research Organization (JACCRO) further conducted the JACCRO GC-07 trial, showing that S-1 plus docetaxel for 6 months and followed by S-1 alone for 6 months is a better choice for stage III GC patients (31, 32). In Western patients, postoperative chemoradiotherapy should be a considered addition for these patients (33). Therefore, the improvement of detecting N3b, which could detect more stage IIIB or IIIC patients, could also make the adjuvant treatment strategy more reasonable and has great clinical significance for appraising prognosis. Overall, the upstaging caused by the N status implies a change in patient treatment (with the indication of adjuvant therapy) and adds greater clinical significance to the present study.

Importantly, surgeons can not only could retrieve more LNs but also divide LNs into stations and count sectioned LNs as a single LN at each station. The status of MLNs at each station could be vital for further investigating the regulation of LN metastasis and elucidating the metastasis model of LNs, both of which are also very important for assessing biological characteristics and making suitable treatment strategies in subsequent research. At the same time, the count of RLNs at each station could also improve quality control in the surgical treatment of GC and promote the implementation of standard D2 radical LN dissection for GC (34).

In our analysis, LN examination by the specialized surgicopathologic team, more advanced pT, tumor size ≥5 cm and combined organ resection were associated with more MLNs. Clearly, T staging, tumor size, and combined organ(s) resection represent the biological characteristics of GC that are related to LN metastasis; this has been widely proven (23, 35–37). The method of LN examination is not associated with the biological characteristics but was still related to the number of MLNs detected, which was mainly due to their impact on the number of RLNs. Given our results, the retrieval of LNs by surgeons immediately after an operation should be the preferred technique over the conventional method by pathologists.

Also, the conventional LN examination by inspection, palpation, and/or serial sectioning is prone to missing very small LNs, and small LNs can also possibly metastasize (38). Noda et al. reported that ignoring small LNs can be a major cause of staging error in GC (38). In his investigation, the mean size of metastatic LNs was 7.80 mm for a total of 23233 LNs. If all LNs with a size of 5 mm or less are ignored when fixed, then 37.8% of all MLNs would have been missed, and downstaging would occur in 14.9 and 4.2% of the cases if all LNs <6 and 4 mm, respectively, were ignored. Therefore, they proposed that all LNs 4 mm or more in size (5 mm when fresh) should be retrieved and examined. Thus, adjuvant technologies are expected to further improve the efficiency of LN examination by harvesting more LNs and detecting smaller LNs on the basis of the conventional LN examination. This method includes LN-revealing solutions and lymphatic tracers.

To detect very small LNs, Koren et al. used LN-revealing solutions to prevent small LNs from being obscured by the surrounding adipose tissue (39). This method yielded LNs significantly smaller than the traditional method (mean size: 3.03 ± 3.43 vs. 6.69 ± 3.43 mm). However, this method required that the entire perigastric fat was carefully detached from the stomach and immersed for 6–12 h in ~3 times its volume of LN-revealing solution, which is a mixture composed of 65 mL of 95% ethanol, 20 mL of diethyl ether, 5 mL of glacial acetic acid and 10 mL of buffered formalin. Subsequently, the fat was washed thoroughly under running tap water and sectioned again at intervals of 2–3 mm. Thus, although this method could significantly increase the number of RLNs and decrease the size of the nodes, its operational program is tedious and time consuming, which makes it difficult to generalize in clinical practice. Subsequently, Carnoy's solution (CS) has been used as a new fat-clearing solution in LN-revealing solutions; however, it also had similar methodological limitations (40). Over decades, lymphatic tracers, including methylene blue, indocyanine green, and the intraoperative radiation technique with a gamma probe, have also been used as guidance for LN searching and dissection (41, 42). However, no ideal materials have been found due to the limitation of their staining efficiency, the relatively complicated lymphatic flow of the gastric system, radiation injury, and expense. Carbon nanoparticles (CN) are one of the most commonly used nanoparticles to trace LNs in some tumors because they are inexpensive and widely available (43–45). Recently, LN labeling with CN was applied to GC and can improve the number of RLNs and the detection of MLNs (46). To evaluate the application value of LN tracing with CN by preoperative endoscopic subserosal injection in laparoscopic radical gastrectomy, Hong et al. randomly assigned patients to a trial group and control group. The results showed that the mean number of RLNs in the trial group was significantly higher than that in the control group (35.5 ± 8.5 vs. 29.5 ± 6.5, *p* < 0.05). Regarding the LNs with and without black dye in the trial group, the rate of MLNs was significantly higher than that in LNs with black dye (17.3 vs. 4.0%, *p* < 0.01) (46). In our center, we use the method of preoperative submucosal injection of CN followed by a conventional LN examination approach in rectal cancer after neoadjuvant chemoradiotherapy. Similarly, a more precise oncologic prognostic assessment is provided by increasing the number of RLNs (21.1 vs. 8.0, *p* < 0.001) using the dye-tracing method. Furthermore, in the CN group, the mean time for LN retrieval was shorter than that in the control group (27.6 vs. 34.6 min, *p* < 0.001) (45). Li et al. conducted a prospective randomized trial to evaluate the efficiency and safety of CN for retrieving LNs in advanced GC (47). In the experimental group, 1.0 mL of CN was injected into the subserosa of the stomach at five points around the tumor about 10 min before open gastrectomy with D2 dissection. The same procedure was performed directly without any coloring material in the control arm. In line with previous studies, the mean number of RLNs was higher in the experimental group than that in the control group (38.33 vs. 28.27, *p* = 0.041). A smaller diameter of LNs was observed in the experimental arm (3.32 vs. 4.30 mm, *p* = 0.023). However, subgroup analysis showed that no additional MLNs were harvested in the experimental group. Nevertheless, the CN approach also has many potential weaknesses that limit its use, regardless of whether 0.5 mL of CN suspension is injected into the submucosal layer using a rectal speculum at 3 points around the primary tumor 1 day before surgery or whether it is injected into the subserosa of the stomach at five points around the tumor about 10 min before surgery, both require highly technical operation, have a steep learning curve, and increase the workload for surgeons. Particularly, the injection of the CN suspension into the submucosal layer around the primary tumor is a highly technical operation, and has the risk of colliding the tumor. Furthermore, the diffusion of CN may affect the judgment of the location of the tumor and the extent of resection during surgery.

Therefore, adjuvant technology has not been widely used in the clinic as the main approach because of its inherent weaknesses. However, adjuvant technology has the potential to help detect more LNs with high efficacy, especially for small LNs, on the basis of routine LN examination relying on the operator's vision and tactile sense to detect LNs. Hence, the interdisciplinary cooperation of clinicians, basic medical researchers and chemical material researchers is expected to facilitate the development of a more accurate and effective new tracer or LN-revealing solutions.

There are also apparent limitations in our study. Although the data in our study were prospectively collected (48), our study was not prospectively designed but retrospectively analyzed. Of course, we tried our best to compensate for this limitation. For example, only standard curative D2 distal/total gastrectomy was considered, and patients with previous gastrectomy (gastric stump cancer) were excluded, as were those who underwent neoadjuvant therapy to control for other surgically-related variables. In addition, as a result of the non-prospective design, the duration of the dissection of each case was not recorded, so the assessment of the two approaches on the prospect of time was not possible. Therefore, the operation duration of each method should be taken into consideration in the design of subsequent RCTs. In addition, the size of the RLNs and MLNs in each group was not registered in our database, so we could not investigate whether the method by the specialized surgicopathologic team could retrieve smaller LNs and detect small MLNs than that by pathologists. Since ignoring small LNs can be a major cause of staging error in GC (38), the size of the RLNs and MLNs should also be recorded and analyzed in the subsequent RCTs.

## Conclusions

The retrieval of LNs immediately postoperatively by the surgicopathologic team in our center could significantly improve the number of RLNs, detect more MLNs, and screen more patients with N3b node status for GC. This method could reduce stage migration and therefore has a significant impact on prognostic evaluation and the formulation of adjuvant therapy strategies.

## Data Availability Statement

The datasets generated for this study will not be made publicly available to protect the patients' privacy.

## Ethics Statement

The data collection protocol was approved by the Ethics Committee of Nanfang Hospital, Southern Medical University. Written informed consent was obtained from all the patients in the study.

## Author Contributions

JY, GL, and HL made substantial contributions to the conception and design, and interpretation of data. XC, YC, YH, TiaL, and JL contributed in drafting the manuscript or critically revising it for important intellectual content. XC, TuaL, TaoL, HH, YZ, TinL, and HC collected and analyzed the data. All the authors approved the final manuscript submitted. Each author participated sufficiently in the work to take public responsibility for appropriate portions of the content and agreed to be accountable for all aspects of the work in ensuring that questions related to the accuracy or integrity of any part of the work are appropriately investigated and resolved.

### Conflict of Interest

The authors declare that the paper was conducted in the absence of any commercial or financial relationships that could be construed as a potential conflict of interest.

## Appendix

The procedures of LNs examination after gastrectomy by the surgicopathologists.

The division of regional fatty tissue-containing LNs into stations was performed by a surgicopathologic team postoperatively within 5 min. To improve the accuracy of perigastric LN substation and the number of LNs detected, vessel clip markers were used on the side of the specimen when severing the important perigastric vessels (left gastroepiploic, right gastroepiploic, left gastric and right gastric arteries, and veins). After the specimens were removed extracorporeally, the anatomical position of the main perigastric vessels was marked and located by a vessel clip. Then, according to the Japanese Convention on the Treatment of Gastric Cancer, the perigastric tissues of the lesser and greater curvatures were subjected to substation disposal. After separation, the LNs were removed from the perigastric tissues by experienced sample handlers through visual and tactile approaches. LNs are mostly distributed along blood vessels, so we should pay attention to protecting the main blood vessels when we examine LNs and retrieve them along blood vessels. LNs are easily confused with fat granules. In color discrimination, fat particles tend to be orange, some LNs tend to white and more transparent. The texture of the LNs is tougher, harder and less fragile. Finally, the LNs were sent to each station in separate bags for pathological examination.
